# COVID-19 control measures unexpectedly increased the duration of stay at High Speed Rail stations during the first community outbreak in Taiwan

**DOI:** 10.1186/s12889-024-17964-6

**Published:** 2024-02-22

**Authors:** Ning Chang, Yi-chen Tsai, Wei J. Chen, Chung-Chuan Lo, Hsiao-Han Chang

**Affiliations:** 1 Institute of Systems Neuroscience, National Tsing Hua University, Hsinchu, Taiwan; 2 Institute of Information Management, National Yang Ming Chiao Tung University, Taipei, Taiwan; 3 Centers of Genomic and Precision Medicine, National Taiwan University, Taipei, Taiwan; 4 Institute of Epidemiology and Preventive Medicine, College of Public Health, National Taiwan University, Taipei, Taiwan; 5 Center for Neuropsychiatric Research, National Health Research Institutes, Zhunan, Miaoli County, Taiwan; 6 Brain Research Center, National Tsing Hua University, Hsinchu, Taiwan; 7 Department of Life Science and Institute of Bioinformatics and Structural Biology, National Tsing Hua University, Hsinchu, Taiwan

**Keywords:** Mobility, Disease control measures, Duration of stay, Number of passengers

## Abstract

During the COVID-19 pandemic, Taiwan has implemented strict border controls and community spread prevention measures. As part of these efforts, the government also implemented measures for public transportation. In Taiwan, there are two primary public transportation systems: Taiwan Railways (TR) is commonly utilized for local travel, while the Taiwan High-Speed Rail (THSR) is preferred for business trips and long-distance journeys due to its higher speed. In this study, we examined the impact of these disease prevention measures on the number of passengers and duration of stay in two major public transportation systems during the first community outbreak from April 29th to May 29th, 2021. Using data from a local telecommunications company, our study observed an expected decrease in the number of passengers after the cancellation of non-reserved seats at both TR and THSR stations across all 19 cities in the main island of Taiwan. Surprisingly, however, the duration of stay in some of the cities unexpectedly increased, especially at THSR stations. This unanticipated rise in the duration of stay has the potential to elevate contact probability among passengers and, consequently, the transmission rate. Our analysis shows that intervention policies may result in unforeseen outcomes, highlighting the crucial role of human mobility data as a real-time reference for policymakers. It enables them to monitor the impact of disease prevention measures and facilitates informed, data-driven decision-making.

## Introduction

Since the first imported case of COVID-19 was reported in Taiwan in January 2020 [1], the government has implemented a range of control measures to reduce the risk of local transmission. These measures include strict border control and the implementation of nonpharmaceutical interventions. Specifically, border control measures include precautionary quarantine and testing for individuals with a high-risk travel history or contact with confirmed cases, with records being documented using the national health insurance card system [2, 3]. Locally, nonpharmaceutical interventions such as social distancing, temperature checks and mask-wearing have been enforced in public transportation, large gatherings, and schools, and hand sanitizers are commonly provided in public places [4].

For policymakers, continuous monitoring and assessment of policy effectiveness and compliance are crucial. Policies and associated public messaging should be updated as needed over time. Survey data is commonly employed to examine responses and behavioral changes following the implementation of control methods. For instance, the CoMix survey gathers information on people’s behaviors in response to COVID-19 across Europe, providing a great source for monitoring the effects of policies [5]. However, survey data collection can be resource-intensive. With the widespread use of cell phones, the aggregated information on human movements from cell phone companies or applications presents an opportunity to assess the effectiveness of policies aimed at promoting social distancing and reducing the probability of contact between individuals in real-time [6]. In Taiwan, the government has launched an app that provides real-time estimates of population density derived from the number of mobile phone users in key tourist locations [7]. Many studies, relying on survey data or open sources like Google Mobility, have demonstrated a significant reduction in human mobility during the COVID-19 pandemic in various countries, including Taiwan [8–12]. However, ongoing compliance monitoring is essential to ensure efficacy. For example, a study conducted in Ghana revealed limited compliance with recommendations such as social distancing and mask-wearing in some transportation stations, underscoring the need for additional support and guidance [13].

In April 2021, Taiwan experienced its first wave of local COVID-19 outbreaks, followed by a second wave in January 2022. During the initial wave of local COVID-19 outbreaks, Taiwan’s major inter-city transportation systems, the Taiwan Railways (TR) and High-Speed Rail (THSR), implemented measures such as canceling standing-room-only tickets, non-reserved seating, electronic tickets, and multi-ride tickets on May 15th, 2021 (Table 1) [14–16]. These measures aimed to prevent non-registered ticket purchasers from entering the carriage, reducing population density, and minimizing contact between strangers inside trains or in the stations. However, human behaviors are influenced by various factors, and the effectiveness of these measures remains to be evaluated. While Google mobility data has been used to illustrate reduced mobility in transit stations, it lacks the granularity to examine mobility patterns in different transportation systems [9]. In this study, we utilized aggregated data from FarEasTone Telecommunications to examine changes in the number of people and the duration of their stay at Taiwan Railways (TR) and High-Speed Rail (THSR) stations across all 19 cities/counties on the main island of Taiwan from April 29th to May 29th, 2021. Our analysis of mobility data provides insights into the effectiveness of the government’s measures in promoting social distancing within the two major inter-city transportation systems in Taiwan.

**Table 1:** Epidemic prevention policies in Taiwan Railways Administration and Taiwan High Speed Rail Corporation from April, 2020 to April, 2022

Month that new policy was applied	Taiwan Railway (TR)	Taiwan High Speed Rail (THSR)
2020/04	- Passengers who developed fever, cough, or other acute respiratory symptoms were refused entry. - Food consumption inside carriages and stops was prohibited. - Passengers were required to wear masks at all times. - Standing-room-only tickets were canceled	- Passengers who developed fever, cough, or other acute respiratory symptoms were refused entry. - Food consumption inside carriages was prohibited. - Passengers were required to wear masks at all times. - Seating was arranged in a checkerboard-like pattern (level 1), ensuring a distance of at least two rows between passengers.
2020/06	- Body temperature checking was required. - Passengers were allowed to remove their masks if they maintained social distance. - Food consumption was allowed. - Standing-room-only tickets were partially open.	- Food consumption inside carriages was allowed.
2020/12	- Passengers were required to wear masks at all times.	
2021/02	- Food consumption inside carriages was prohibited. - The selling of standing-room-only tickets was restricted.	- Non-reserved seats were canceled during Chinese new year and spring break. - Periodic tickets, multi-ride tickets, and e-tickets were unavailable.
2021/05	- Standing-room-only tickets for long-distance and limited express with reserved seats were canceled. - Contact tracing was initiated.	- Non-reserved seats were fully canceled. - Contact tracing was initiated. - The checkerboard-like seating was upgraded to level 2 by increasing the distance between passengers by more rows of chairs. - Food consumption inside stations was prohibited. - Food consumption inside carriages was prohibited after 8 p.m.
2021/06		- The checkerboard-like seating was upgraded to level 3. - Food consumption inside carriages was allowed.
2021/07		- Ticket sales were limited to 70%. - The checkerboard-like seating was downgraded to level 2. - Food consumption inside stations was allowed.
2021/08		- Ticket sales were limited to 80%. - The checkerboard-like seating was canceled.
2021/11		- Non-reserved seats were made available for sale. - Food consumption inside carriages was allowed.
2021/12	- Standing-room-only tickets were made available for sale.	
2022/01		- Food consumption inside carriages was prohibited.
2022/03		- Food consumption inside carriages was allowed.
2022/04	- Contact tracing was canceled.	- Contact tracing was canceled.

## Methods

Our study utilized data from two sources: Google’s Community Mobility Reports [17] and FarEasTone Telecommunications Co., Ltd., which is one of the major telecommunications companies in Taiwan, with a market share of approximately 25% [18]. The data from FarEasTone Telecommunications provide information on the relative changes in the number of clients and their duration of stay in TR and THSR stations during the period from April 29th to May 29th, 2021. The relative changes represent the percentage changes compared with the baseline values. The baseline values for weekdays and weekends were calculated separately. For weekdays, the baseline value was obtained by taking the average among February 6 (Thursday), 10 (Tuesday), and 11 (Wednesday), 2020. For weekends, the baseline value was the average of February 8 (Saturday) and 9 (Sunday), 2020. We examined the number of passengers and their duration of stay at 19 TR stations and 11 THSR stations located across 19 cities/counties in Taiwan.

We analyzed the relative changes in the number of clients in transit stations, which encompassed subway stations, sea ports, train stations, and taxi stands, from Google’s Community Mobility Reports [17]. The baseline values for the Google data were established using the median values from the corresponding day of the week between January 3rd and February 6th, 2020. The relative changes were also calculated by comparing the values during the study period with the baseline values.

The number of cases in Taiwan was downloaded from the website of the Centers for Disease Control in Taiwan [19].

## Results

**Fig. 1:**
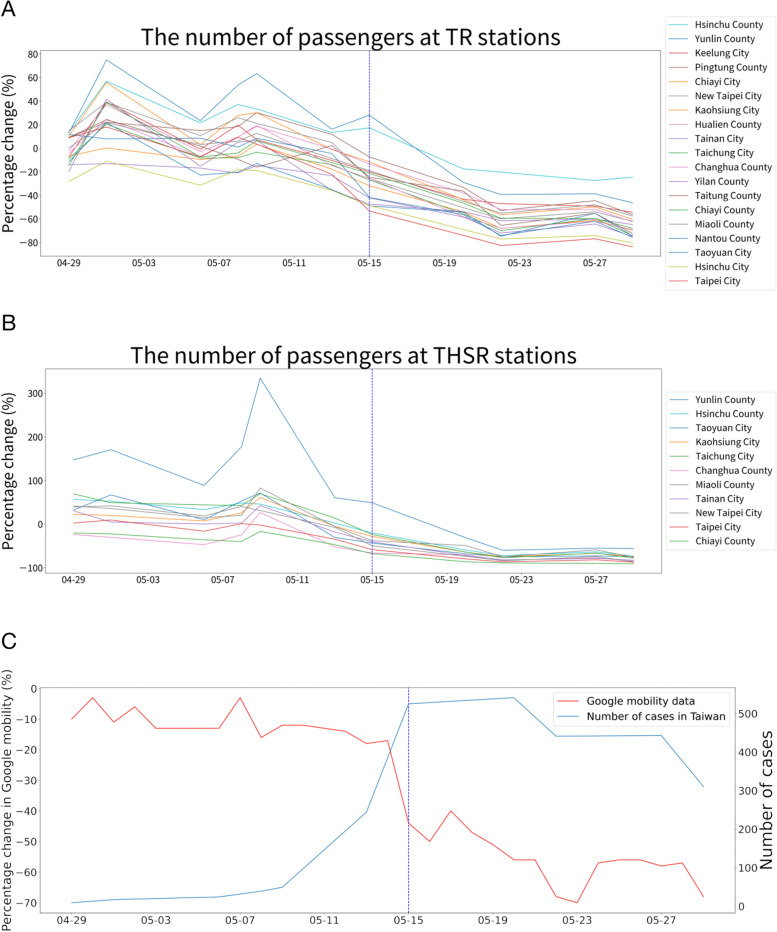
Relative changes in the number of passengers on public transportation stations over time. The blue dotted line indicates the date when the cancellation of non-reserved seats began (May 15th, 2021). **A** The percentage changes in number of passengers at TR stations in 19 cities from April 29th to May 29th, 2021, based on the data provided by FarEasTone Telecommunications are shown. The number of passengers tended to decrease over time. **B** The percentage changes in number of passengers at 11 THSR stations during the same time period, based on data from FarEasTone Telecommunications, also indicate a decreasing trend. **C** Data from Google’s Community Mobility Reports (red line) also show a decreasing trend in the number of passengers in transit stations during the same time period. The number of daily new cases (blue line) in Taiwan increased

By examining population mobility data at TR and THSR stations across all 19 cities/counties, we observed a consistent decline in passenger numbers at both types of stations during Taiwan’s first wave of local outbreaks. (Fig. 1A, B). Moreover, Google mobility data indicated a similar reduction in visitor numbers at transit stations during the same period (Fig. 1C). These results collectively suggest that the disease control measures implemented in transportation systems were effective in reducing passenger numbers.

**Fig. 2:**
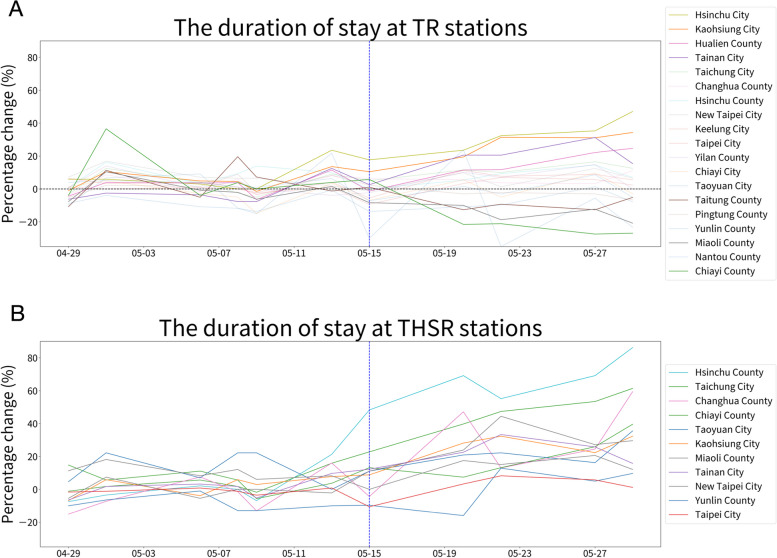
Relative changes in the duration of stay on public transportation stations over time. The blue dotted line indicates the date when the cancellation of non-reserved seats began (May 15th, 2021). **A** The percentage changes in the duration of stay at Taiwan Railways (TR) stations in 19 cities from April 29th to May 29th, 2021, based on the data provided by FarEasTone Telecommunications are shown. The trend varies among cities. The cities with the top four increasing trends (Hsinchu City, Kaohsiung City, Hualien County, and Tainan City) and the top three decreasing trends (Taitung County, Miaoli County and Chiayi County) in the percentage change of the duration of stay were displayed with lines of less transparent colors. **B** The percentage changes in the duration of stay at 11 Taiwan High-Speed Rail (THSR) stations during the same period, based on the data provided by FarEasTone Telecommunications, indicate an increasing trend after the cancellation of non-reserved seats

However, unexpectedly, the duration of stay at most THSR stations showed an increasing trend, whereas patterns in TR stations varied across cities (Fig. 2). Among all THSR stations, Hsinchu County and Taichung City showed the most significant increase in the duration of stay. For TR stations, Hsinchu City, Tainan City, Kaohsiung City, and Hualien County exhibited a significant increase to 13 to 47% on May 15th in; conversely, Miaoli County, Chiayi County, and Taitung County displayed a significant downward trend to − 4% to − 26% (Fig. 2A).

## Discussion

In summary, while the epidemic prevention policies were successful in reducing the overall number of passengers and contact probability inside train carriages, they inadvertently led to an increase in the duration of stay and potential contact probability within station waiting areas. This unanticipated finding highlights the importance of analyzing human mobility data to monitor the effects of disease prevention policies and continuously adapt them accordingly.

Taiwan Railways (TR) and the Taiwan High-Speed Rail (THSR) are the two primary public transportation systems for intercity travel in Taiwan. Each city has at most one THSR station [20], and THSR is particularly popular for business and long-distance travel due to its higher speed. Differently, there are 241 TR stations in Taiwan, and Taiwan Railways is frequently used for local travel. The variation in the pattern of the duration of stay in TR stations among cities/counties found in this study may be attributed to factors such as the uneven distribution of stations, differing travel types, variations in population densities, and passenger volumes.

The observed increase in the duration of stay at most THSR stations could be attributed to the cancellation of non-reserved seating, which began on May 15th, 2021. Following this change, a more pronounced trend towards longer station stays was noted. This could be due to longer waiting times resulting from the seating policy change. Non-reserved seating primarily serves commuters, and the notably higher increase in duration of stay in Hsinchu aligns with its larger commuter population using THSR services [21]. Furthermore, the THSR stations in Hsinchu and Taichung, not being located in city centers and lacking nearby alternative intercity transportation options, likely cause passengers to wait for the next available train rather than seeking other transportation methods. This situation potentially leads to extended waiting times, especially after the cancellation of non-reserved seating. However, this phenomenon at the Hsinchu and Taichung stations cannot be solely attributed to population density or user numbers, as these factors are highest in Taipei City [22, 23].

Due to the nature of this observational study, we were unable to test the causal relationship between the cancellation of non-reserved seating and the increased duration of stay in the THSR stations. Several individual-level factors, including personal perceived risk, vaccine, or infection status, could potentially influence the duration of stay. A survey study collecting these data could be instrumental in considering these factors collectively. Similarly, the consistent reduction in the number of passengers across both types of transportation systems in all cities can potentially be attributed to the overall increase in COVID-19 cases in Taiwan (Fig. 1C). The impact of disease prevention policies targeting transportation systems and the general increase in cases are confounding factors that cannot be distinguished in our study.

Public transportation has been recognized as a potential hotspot for COVID-19 transmission [24]. Governments in many countries have implemented COVID-19 regulations for public transportation systems to mitigate the risk of transmission [8, 25–30]. These measures include social distancing (such as increased spacing between seats, online booking, and reducing the number of passengers on each train), mask-wearing, and surface disinfection, among others. Additionally, studies have explored optimal seat assignments to enhance social distancing [31, 32], and models have been developed to estimate passenger flow and density [33, 34]. While the general association between mobility and the stringency of government restrictions has been shown [35], research on the effects of various transportation policies in response to COVID-19 is still lacking. In this study, we separately analyzed mobility patterns in two public transportation systems and examined the number of passengers and the duration of stay in stations. This approach enabled us to reveal differences between the two transportation systems and two aspects of human mobility in response to policy changes. We provide an example illustrating the unexpected increase in the duration of stay after the implementation of the policy that cancels non-reserved seating, highlighting the importance of monitoring policy impacts since unintended outcomes may occur. Given that compliance and responses to COVID-19 regulations may vary over time [36], even if the policies remain the same, setting up a streamlined system for continuous monitoring is recommended for future disease control. To mitigate the potential impact of increased station stay duration on disease transmission, measures such as prohibiting food consumption in stations, providing real-time train capacity information, reserving part of the available seats for vulnerable users, and increasing train frequency can be considered [37–40].

## Conclusion

To understand the impact of disease prevention methods on social distancing in public, we analyzed two aspects of human mobility at public transit stations— the number of passengers and the duration of stay—using data from FarEasTone Telecommunications Co. and Google’s Community Mobility Reports. Our findings reveal a decrease in the number of people in public transportation stations following the implementation of control measures, but an unexpected increase in the duration of stay, which might indirectly increase the contact probability in stations. This study underscores the importance of using human mobility data to regularly monitor the effectiveness of policies aimed at reducing the spread of disease. By providing insights into the behavior of individuals in response to public health measures, such data, in conjunction with other factors like population density and vaccine coverage, can aid in shaping the development of more effective intervention policies to mitigate the spread of infectious diseases.

## Data Availability

The data that support the findings of this study are available from Far EasTone Telecommunications (EFT). Restrictions apply to the availability of these data, which were used under the data use agreement with EFT for this study. Data are available from the authors with the permission of EFT.
